# Epidemiological, clinical characterization and treatment patterns of migraine patients in a Colombian cohort from 2018 to 2022

**DOI:** 10.1186/s10194-024-01918-9

**Published:** 2024-12-24

**Authors:** A. C. Rubio, J. A. Arciniegas, J. E. Bolanos-Lopez, F. J. Gonzalez, D. Gomez, A. Mesa, C. Bello, M. Garcia, L. E. Perez, J. M. Reyes

**Affiliations:** 1 Pfizer SAS, Bogota, Colombia; 2 Suramericana IPS, Medellin, Colombia

**Keywords:** Migraine, Incidence, Prevalence, Colombia, Treatment patterns, Cardiovascular diseases

## Abstract

**Background:**

To describe the epidemiology and clinical characteristics of migraine and the status of treatment in Colombia. Additionally, the use of health resources by patients was measured.

**Methods:**

This was a non-interventional, retrospective, descriptive study conducted in one Colombian Health Management Organization (HMO) from 2018 to 2022 with a follow-up period of 5 years. Migraine patients were identified using the International Code Disease 10th version G43, and the diagnosis was confirmed by a neurologist. The first recorded migraine diagnosis was defined as the index date. Medical records, claim databases and other electronic databases from the HMO were used to determine the clinical characteristics, treatments, and health care services.

**Results:**

A total of 89,227 patients were included in the study. The mean follow-up period was 3.7 years (standard deviation 1.2). Most of them were women (84.9%). Many patients were first seen by a general practitioner (82.6%), and only 8.9% were first seen by a neurologist. The prevalence of migraine during follow-up was between 1.69 and 2.42 patients in 100 HMO affiliates in 2020, the year with the highest prevalence (2.42 [95% CI 2.41–2.44]), and the incidence ranged from 0.032 to 1.72 per 100 patient-year at risk of developing migraine. Hypertension (21.3%), arrythmia (4.1%) and structural heart disease (3.4%) were the most common cardiovascular diseases. The annual mean number of outpatient consultations in 2018 was 1.43 consultations per patient, which decreased to 0.68 in 2022. The most frequent treatments for acute events were nonsteroidal anti-inflammatory drugs (NSAIDs) (range 37-42%) in monotherapy, combinations of analgesics (range 14-35%), and corticosteroids (range 10-15%). Triptans were used in 4% of patients in the first medication record, reaching a maximum of 16% of patients. Among preventive treatments, beta-blockers (24-49%) and antiepileptics (29-41%) were the most common.

**Conclusion:**

The prevalence of migraine in Colombia according to health electronic databases was lower than that reported in previous studies conducted in the country. The treatment patterns for acute and preventive treatment of migraine follow the recommendations of different guidelines. Cardiovascular disease is relevant for the management of migraine.

**Supplementary Information:**

The online version contains supplementary material available at 10.1186/s10194-024-01918-9.

## Background

Migraine is a disabling primary headache disorder [1] characterized by headaches and associated symptoms that can lead to considerable disruption of the professional and private lives of affected individuals. According to the GBD Study 2019, migraine alone was the second most common cause of disability and first among women under 50 years of age [2]. Although migraine is a common disorder in urban communities in Latin America, the limited data on the prevalence of migraine in many countries are remarkable, possibly related to the lack of resources, underdiagnosis of migraine, and the limitations of study groups or epidemiologists with interest in this disease. Not all countries in Latin America have population-based epidemiological studies of migraine, and most of these studies were conducted many years ago, with few patients participating [3]. A meta-analysis of population-based studies in Latin America and the Caribbean reported that the prevalence of migraine was 15.03% (95% CI: 12.04–18.29%; I2 = 99.01%), with a greater effect on females [4]. The global prevalence peaks between the ages of 25 and 55 [5].

The diagnosis of migraine was based on clinical history and the exclusion of secondary headache according to the International Classification of Headache Disorders (ICHD) criteria. In the 3rd Edition of the ICHD [1], migraine is classified into three main types: migraine without aura, migraine with aura, and chronic migraine. Currently, migraine treatment is individualized based on patient preference, clinical features and type of migraine, episode-related disability, the presence of coexistent illness or contraindications (e.g., cardiovascular disease) and prior treatment response and concomitant medications [5, 6]. However, it has been reported that few people with migraine consult physicians, and migraine-specific medications are used inadequately even among those who do. In one study, only 25% of patients with chronic migraine received a correct diagnosis, and only 4.5% of individuals consulted a health care professional for migraine, received an accurate diagnosis, and were prescribed minimal acute and preventive pharmacological treatments [7].

The treatment of patients with migraine aims to relieve pain or limit attacks, reduce disability, restore function, improve health-related quality of life and manage comorbidities. Pharmacological therapy comprises both acute and preventive treatments. The development and emergence of novel medications, device technologies, and novel formulations of established drug therapies have led to much-needed advances in the acute and preventive treatment of migraine [5]. Acute treatment options include analgesics (the most common nonsteroidal anti-inflammatory drugs [NSAIDs]), antiemetics (e.g., metoclopramide), triptans (e.g., oral or subcutaneous sumatriptan), emergency medications (e.g., intravenous metoclopramide and subcutaneous sumatriptan), calcitonin gene-related peptide (CGRP) receptor antagonists (e.g., ubrogepant and rimegepant), and serotonin (5-HT1F) agonists (e.g., lasmiditan). Preventive medications are primarily used to reduce the frequency of episodes and include an intravenous anti-CGRP ligand monoclonal antibody (e.g., eptinezumab) [6, 8, 9]. In addition, nonpharmacologic therapy, such as neuromodulation, biobehavioral treatment, and nutraceuticals, is available for both acute and preventive treatment and may be used alone or in combination for the management of migraine [6].

Due to the limitations of population-based epidemiological studies of migraine and the emergence of novel medications, the development of research and analyses on local data on migraine are important and robust sources of information to support decision-making for health care stakeholders, researchers, and policy-makers. The main purpose of this study was to provide updated information on the epidemiology and clinical characteristics of migraine and the status of treatment in Colombia. Additionally, the results of this study are intended to describe the use of health resources by patients from the time they are diagnosed with migraine.

## Methods

### Study design and data source

This was a retrospective, observational study of a Colombian Health Management Organization (HMO) during the 5-year period from 2018 to 2022. The HMO, SURA, was characterized as the third largest health insurer in Colombia, with approximately 5.4 million people (approximately 10.4% of the population) [10]. The population included approximately 3.3 million insureds (≥ 18 years) over the 5-year study period.

The databases contain longitudinal information on insured individuals with respect to all areas of services administered by the HMO. The HMO includes detailed data on inpatient and outpatient care, diagnosis, demographic characterization and claim databases based on the International Classification of Diseases, 10th revision (ICD-10). This study only used secondary data collected through electronic health resources built by the HMO. All data were subjected to a cleaning process in which the following four aspects were evaluated by the HMO: consistency, completeness, validity, and uniqueness. Access to the data are strictly regulated, and authorization for its extraction was requested by the HMO under national data privacy standards. The databases were anonymized and deidentified before analysis.

### Characteristics of the participants

The study period for data collection was January 01, 2018, to December 31, 2022. Patients and inpatients (aged at least 18 years) with migraine were identified according to inclusion criteria based on the recorded medical diagnosis using the G43 code established by the ICD-10 (G43.0, G43.1, G43.2, G43.3, G43.8, G43.9) and confirmation by a neurologist or another treating physician.

In the first step, patients with migraine according to the ICD-10 were identified in the HMO database. Then, for diagnosis confirmation, the diagnosis made by a neurologist was validated in the first record, or if it was made by a physician other than a neurologist, the migraine diagnosis was also confirmed by neurologist. The diagnosis of migraine in Colombia mainly followed the criteria of the ICHD, 3rd Edition. Patients with other comorbidities documented to be able to cause headache, headaches related to trauma or other systemic diseases were excluded. For each year of the study period, the prevalence and incidence rate were calculated based on the HMO-insured population estimated by sex and age. An incident case was defined as a patient with a first diagnosis of migraine after one year of the index date.

In the second step, the study included eligible outpatients and inpatients diagnosed with migraine from January 01, 2018, to December 31, 2021, who, in the second step, had continuous insurance in the HMO from the index date until at least one year of follow-up. The patients were followed to characterize the concurrent diagnoses, treatment patterns, and use of health resources. The first recorded migraine diagnosis was defined as the index date. Follow-up (time period subsequent to the index date) was variable in length, with each person followed up until (i) discontinuation of continuous HMO insurance, (ii) death, or (iii) the end of the study period (31 December 2022), whichever was earliest.

### Data collection

Demographic characteristics (age and sex) were obtained in the index year. Age was categorized into seven groups (18–24, 25–34, 35–44, 45–54, 55–64, 65–74 and ≥ 75 years). Migraine diagnoses and comorbidities, specialty of the health care professional who provided care, migraine treatments (medication records of type and number), and hospitalization data were collected throughout the study period. Cardiovascular risk factor data, as described in the ICD-10 specific code (Supplementary Table 1), were collected from the initial migraine diagnosis.

Acute migraine therapy was defined as medication prescribed for migraine management during the period of the migraine episode. Preventive migraine therapy was defined as preventive only if a diagnosis of migraine was identified within the same quarter in the in- or outpatient prescription or if the diagnosis of migraine was associated with the prescription. We associated acute/prevention medications with migraine if the prescription was associated with a visit with a G43.xx diagnosis code. The treatments were classified according to the recommendations of local and international guidelines [3, 11, 12]. Discontinuation of medication was defined as the date of the first occurrence of either a switch to another medication for migraine or the end of prescription or if a follow-up prescription was not identified within 90 days of the estimated date of the next prescription of the same medication. For the persistence analysis, the first line could start on the first claim record or in any of the following, as in the first record, patients could have been prescribed acute or preventive therapy.

The patient journey was determined by the health professional group during the initial diagnosis and follow-up, including the number of visits to health care outpatient visits with a general practitioner, the number of visits to outpatient visits with a specialist, the number of visits to emergency care and the number of hospitalizations every following year. These numbers were divided by the number of patients with a diagnosis in the index date year for each year of follow-up to determine the proportions of patients experiencing each event.

### Statistical analysis

The period prevalence was estimated using the study population of the HMO in each year of study. In each age and sex stratum, the number of observed migraine patients (with a first episode or first diagnosis of migraine) each year was calculated over the total number of insured patients (the average number of patients insured by the HMO each year). The annual migraine incidence rate per 1,000 adults was calculated using the total number of incident cases in the at-risk population for each year. The population of patients at risk of migraine comprised all subjects who did not have a diagnosis of migraine at the time of enrollment in the study.

Comorbidities of special interest were those that were contraindications or involved special warnings and precautions for the use of approved preventive/acute medications based on the study of Roessler et al. [13]. The proportions of patients with different migraine subtypes, prescriptions for migraine-specific medications and other medications, and use of health resources were calculated for the study period.

All analyses were descriptive and were performed with appropriate statistical methods using R 4.3.1 [14]. Categorical variables are presented as the number and percentage of patients; continuous variables are summarized as the mean and standard deviation (SD).

## Results

### Sample population

**Fig. 1:**
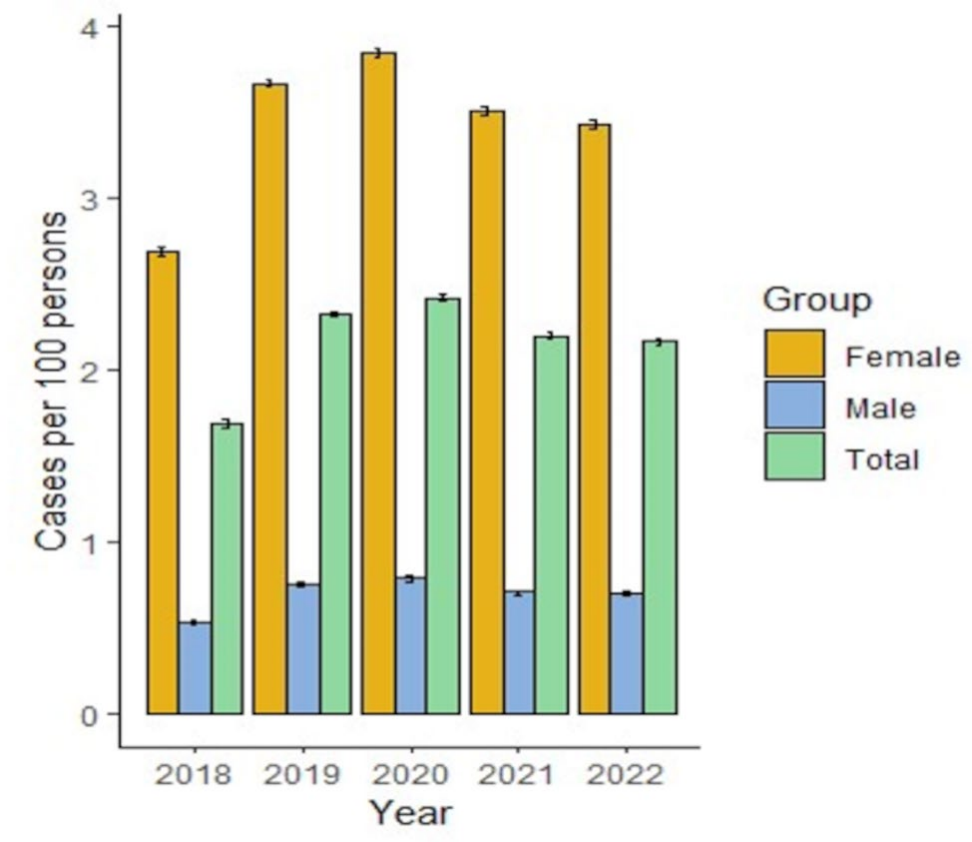
Administrative prevalence of migraine during the period of the study in Colombia discriminating by sex

During the study period, an average of 3,351,441 HMO-insured patients (≥ 18 years old) within the Colombian territory were identified. Of these, 89,227 patients had a confirmed diagnosis of migraine and were included in the study (the study flow is displayed in Fig. 1S). Most patients were female (*n* = 75,726; 84.9%). The mean (SD) age of migraine onset was 38.1 (SD 16.0) in men and 36.8 (SD 13.7) in women, and 55.1% were aged 25 to 44 years (Table 1).

The first recorded diagnosis of migraine, unspecified migraine, was documented for the majority of patients (49.0%), followed by other migraine, complicated migraine, migraine without aura, migraine with aura, and status migrainosus. The characteristics of the population for each type of G43 diagnosis at the time of first diagnosis are also described (Supplementary Table 2).

The follow-up period from the initial diagnosis was 3.7 years (SD 1.2) for both men and women, with a record of between 2 and 9 health care visits by different health professionals (including the initial diagnostic visit).

**Table 1:** Characteristics of population with migraine from database 2018 to 2022

	Category	Total *n*=(89,227)	
		*n*	%
**Sex**	Female	75,726	84.9
	Male	13,501	15.1
**Age**,** total**	18–24	16,918	19.0
	25–34	30,086	33.7
	35–44	19,080	21.4
	45–54	12,173	13.6
	55–64	6,497	7.3
	65–74	2,786	3.1
	≥ 75 Years	1,687	1.9
**Type of health insurance regime** ^**a**^	Contributive	81,859	91.7
	Subsidized	7,368	8.3
**Geographic region of medical care** ^**b**^	Andean/Central	69,515	77.9
	Metropolitan	8011	9.0
	Caribbean	6526	7.3
	Pacific	5175	5.8
**First diagnosis recorded by subtype (ICD-10 code)**	G43.9 Migraine, unspecified	43,747	49.0
	G43.8 Other migraine	14,542	16.3
	G43.3 Complicated migraine (including chronic migraine)	10,666	12.0
	G43.0 Migraine without aura (common migraine)	10,166	11.4
	G43.1 Migraine with aura (classical migraine)	5,594	6.3
	G43.2 Status migrainosus	4,512	5.1

^a^ Type of affiliation to the General System of Social Security in Health (SGSSS in Spanish abbreviation) in Colombia: Contributive regime (it provides mandatory coverage to workers in the formal sector), and Subsidized regime (it covers the low-income population that does not have the capacity to contribute to health system)

^b^ Colombian Region of medical care on index date. Andean region: Antioquia, Boyacá, Caldas, Bogotá D.C. and Cundinamarca, Huila, Quindío, Risaralda, and Santander (no data: Norte de Santander, and Tolima); Caribbean region: Atlántico, Bolívar, Córdoba, and Magdalena (no data: Cesar, La Guajira, Sucre, and San Andres); Pacific Region: Valle del Cauca, Cauca and Nariño (no data: Chocó); Metropolitan region: Bogotá D.C. and Cundinamarca; Orinoco and Amazon region without insured population

**Abbreviations**: n, number of individuals; ICD-10, International Classification of Diseases, tenth revision

### Prevalence and incidence

The prevalence of migraine during follow-up was between 1.69 and 2.42 in 100 HMO affiliates in 2020, the year with the highest prevalence (2.42 [95% CI 2.41–2.44]). The prevalence was higher in women than in men, as shown in Fig. 1. Patients aged 18–24 years and 25–44 years were more prevalent than those of other age groups (between 2.96 and 4.03 years and between 2.46 and 3.42 years, respectively) (Supplementary Table 3).

**Fig. 2:**
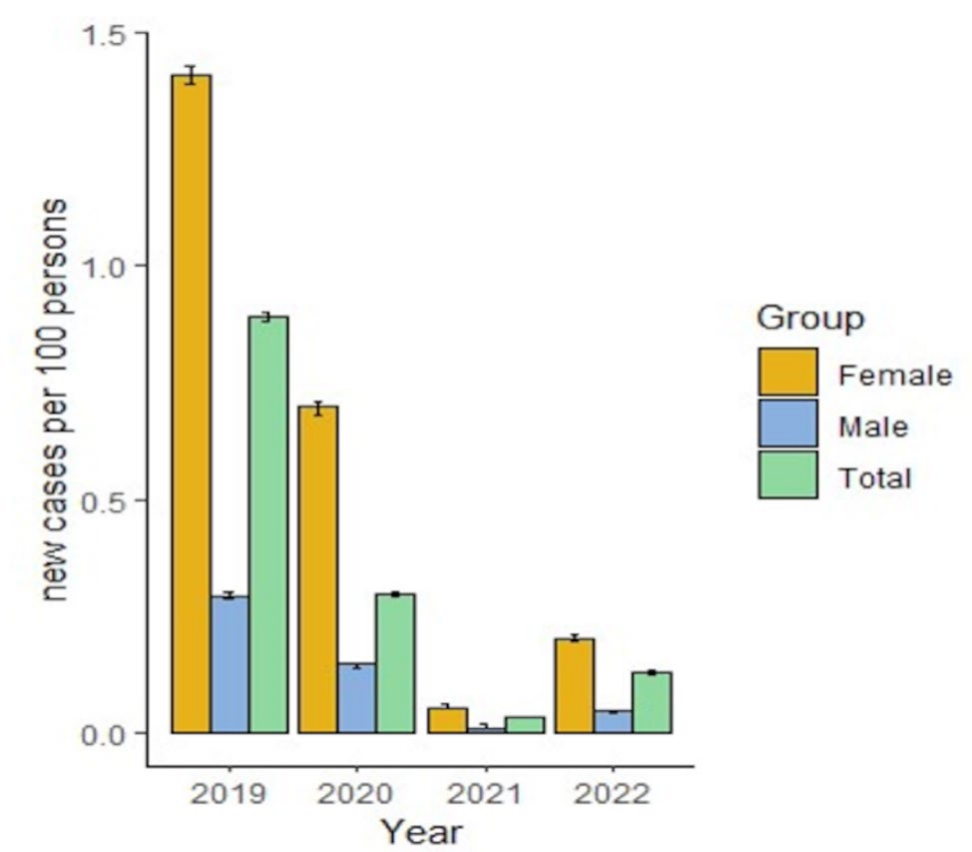
Administrative incidence of migraine during the period of the study in Colombia discriminating by sex

The incidence of migraine showed a decreasing trend from 2019 to 2021, with a mild increase in 2022. The incidence ranged from 0.032 to 1.72 per 100 patients at risk of developing migraine. The lowest incidence was reported in 2021, with 0.032 (95% CI 0.031–0.035) cases per 100 patients. The prevalence of migraine was likely higher in women and patients aged between 18 and 44 years (Supplementary Table 4, Fig. 2).

### Cardiovascular risk or special interest frequency

The most frequent cardiovascular disease in migraine patients was essential hypertension (21.3%). Essential hypertension was most frequent in men and occurred in 24.9% of patients with migraine. In people aged 55–74 years with migraine, essential hypertension was found in 61.8%, while in those aged ≥ 75 years, it was found in 88.3%. Ischemic heart disease and arrythmia were diagnosed in 4.2% and 4.1%, respectively, of the participants in the previous age group. Ischemic heart disease was almost twice as common in men than in women. Ischemic cerebrovascular diseases and heart failure had the same frequency of 2.1%, which was more frequent in men (Table 2). For persons older than 55 years, more than 10% had essential hypertension, pure hypercholesterolemia, type 2 diabetes mellitus, type 1 diabetes mellitus, heart failure, and chronic ischemic heart disease. Most of these conditions are warnings or precautions for the use of triptans, ergotamine derivates, pregabalin, beta-blockers, and amitriptyline. In addition to those previously mentioned, other diseases, such as syncope or collapse (4.1%), angina pectoris (1.6%), cerebral infarction (0.6%), acute myocardial infarction (0.7%), atrial fibrillation (0.7%), and arterial embolism (0.6%), are classified as warnings or precautions for some migraine treatments (Supplementary Table 6).

**Table 2:** Cardiovascular risk factors in study population with migraine from database 2018 to 2022

Cardiovascular conditions	Female		Male		Total	
	No	%	No	%	No	%
Cerebrovascular/cardiovascular disease						
Essential hypertensive	15,629	20.6	3357	24.9	18,926	21.3
Ischemic heart diseases	2737	3.6	1009	7.5	3746	4.2
Ischemic cerebrovascular diseases	1399	1.9	460	3.4	1859	2.1
Peripheral artery diseases	713	0.9	147	1.1	860	1.0
Other significant cardiovascular diseases						
Arrythmia	2948	3.9	693	5.1	3641	4.1
Heart Failure	1404	1.9	476	3.5	1880	2.1
Cardiac surgery and/or implants	96	0.1	45	0.3	141	0.2
Other cardiac conditions	3830	5.1	641	4.7	4471	5.0

### Medication use

**Fig. 3:**
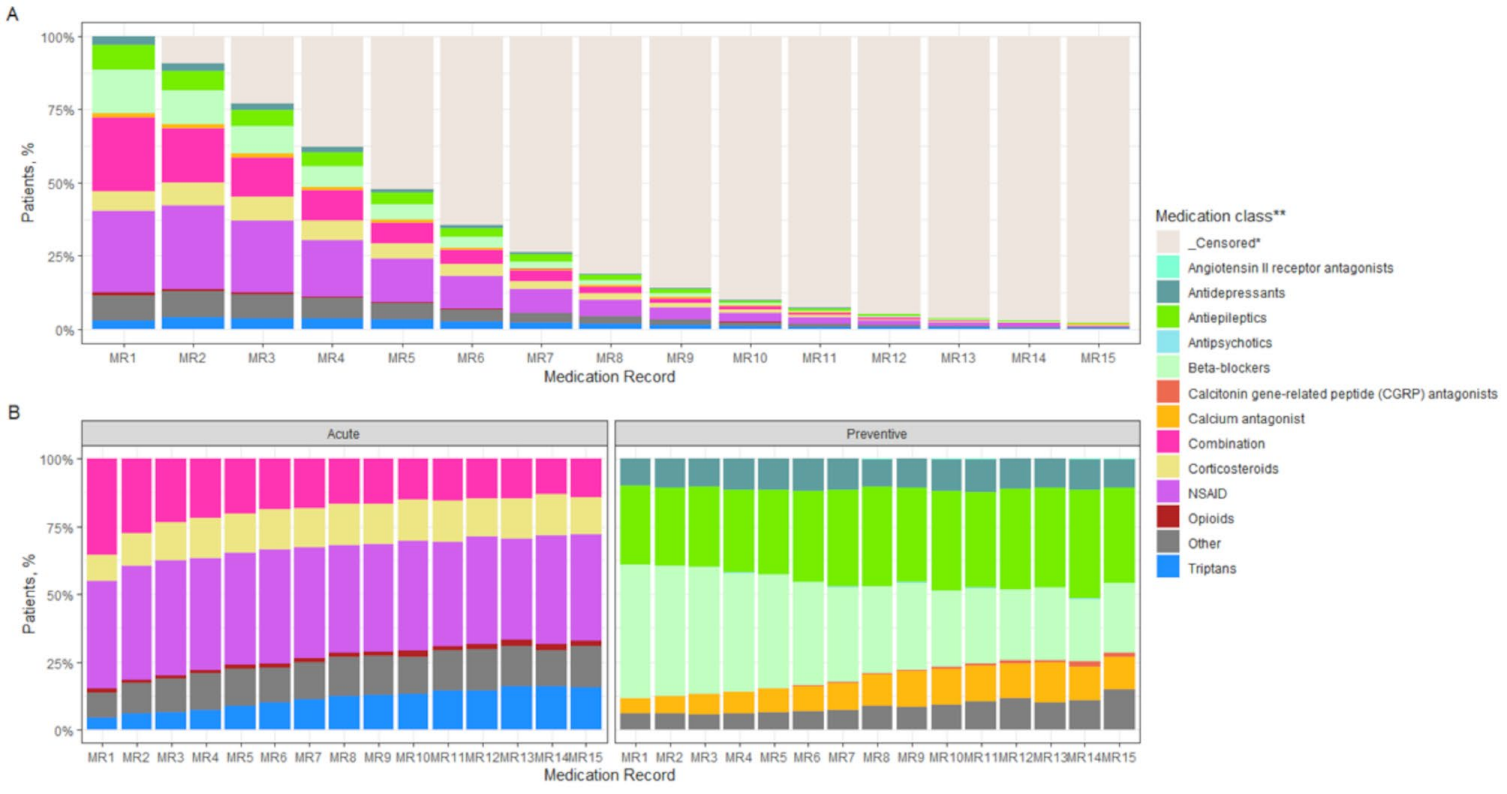
Utilization of medication at the initial diagnosis and follow-up. **A)** All migraine population; **B)** Acute and preventive treatments in the prescribed migraine population. * Censored (Patients with no medication records during subsequent follow-up). ** Medication class: Described in table 3

Overall, 90.2% (80,481/89,227) of the migraine population received at least one prescription for acute or preventive medications of different classes between 2018 and 2022, with a range of 1 to 15 claims observed (Table 3; Fig. 3). The proportions of patients prescribed any acute or preventive medication were 92.4% and 71.8%, respectively, of the total patients with claim data.

Among acute medications, the most frequent were NSAIDs (naproxen, diclofenac and ibuprofen) and corticosteroids. NSAIDs were the most commonly used treatment during the follow-up period for 37–42% of patients. The use of corticosteroids among all patients increased from 10 to 15%. Triptans were used in 16.7% of the study population at any time of the follow-up, with sumatriptan and naratriptan being the most common medications. The use of triptans in the first claims started at 4%, but it progressively increased to 15% during the follow-up. A combination of treatments was used at any time in the study for 65.3% of the patients; however, their use decreased significantly over time. Opioids were reported in between 1% and 3% of the population during the study. A total of 4.9% of patients used opioids at least once during follow-up, with tramadol being the most common. Acetylsalicylic acid, diclofenac, naproxen, dexamethasone and tramadol were used in 73% of the claims for people older than 55 years old. Opioids were used in 15% of the claims for this population.

The most commonly used preventive medications were beta-blockers (specifically, propranolol), antiepileptics (mainly topiramate and valproic acid) and antidepressants (specifically, amitriptyline). Beta-blockers were used at least once in 47.7% of patients, but their use decreased from 49 to 24% during the follow-up. In contrast to the use of beta-blockers, the use of antiepileptics increased from 29 to 41%, and the use of calcium antagonists increased from 6 to 12%. Flunarizine and onabotulinum toxin A were also frequently used (approximately 6% each). The use of onabotulinum toxina A increased from 6 to 15%. CGRP monocolonal antibodies were used in only 0.2% of the population. In adults older than 55 years, propranolol, topiramate, valproic acid, pregabalin, amitriptyline and onabotulinum toxin A were used in 69% of the population.

**Table 3:** Percentage of patients with at least one medication for study population with migraine record by preventive and acute medications

Class (*N* = 80,481) Medications, *n*(%)	Treatment indication	
	Acute	Preventive
**NSAID** *n*** = 58,555 (72,8%)**		
Naproxen	42,246 (72.1)	-
Diclofenac	38,080 (65.0)	-
Ibuprofen	13,637 (23.3)	-
Acetylsalicylic acid	1,040 (1.8)	-
Celecoxib	403 (0.7)	-
Dexketoprofen	25 (0.04)	-
Ketoprofen	12 (0.02)	-
Metamizole/Dipyrone	3 (0.01)	-
**Corticosteroids ***n*** = 31**,**136 (38**,**7%)**		
Dexamethasone	30,766 (98.8)	-
Prednisone	646 (2.1)	-
**Triptans ***n*** = 13**,**446 (16.7%)**		
Sumatriptan	8,644 (64.3)	-
Naratriptan	6,691 (49.8)	-
Zolmitriptan	1,355 (10.1)	-
Eleptriptan	390 (2.9)	-
**Opioids ***n*** = 3**,**951 (4.9%)**		
Tramadol	3,719 (94.1)	-
Dihydrocodeine/Hydrocodone	247 (6.3)	-
Oxycodone	2 (0.1)	-
Combination* *n*** = 52**,**553 (65.3%)**		
**Beta-blockers ***n*** = 38**,**359 (47.7%)**		
Propranolol	-	37,865 (98.7)
Metoprolol	-	748 (1.9)
Bisoprolol	-	2 (0.005)
**Antiepileptic drugs ***n*** = 22**,**554 (28**,**0%)**		
Topiramate	-	11,973 (53.1)
Valproic acid	-	11,494 (51.0)
Divalproex sodium	-	2,082 (9.2)
Pregabalin	-	716 (3.2)
Gabapentin	-	432 (1.9)
**Antidepressants ***n*** = 10**,**596 (13**,**2%)**		
Amitriptyline	-	9,754 (92.1)
Venlafaxine/Desvenlafaxine	-	920 (8.7)
**Calcium antagonist ***n*** = 6**,**762 (8.4%)**		
Flunarizine	-	6,762 (8.4)
**CGRP monoclonal antibodies ***n*** = 131 (0**,**2%)**		
Galcanezumab	-	110 (84.0)
Erenumab	-	29 (22.1)
**Angiotensin II receptor antagonists ** *n* ** = 65 (0.1%)**		
Candesartan	-	65 (0.1)
**Antipsychotics ** *n* ** = 23 (0.03%)**		
Olanzapine	-	23 (0.03)
**Other ***n*** = 32**,**563 (40.5%)**		
Metoclopramide	26,860 (82.5)	-
Onabotulinum toxin A	-	5,002 (15.4)-
Dimenhydrinate	3,019 (9.3)	-
Lisinopril	-	1 (0.003)
Domperidone	-	60 (0.2)
Paracetamol	2 (0.01)	-

- no medication registrations for this indication

* Ergotamine combined with caffeine/lysine clonixinate; Bisoprolol combination amlodipine/thiazides; NSAID with NSAID, muscle relaxants, paracetamol and/or combinations excl. Psycholeptics; Candesartan and/or amlodipine/diuretics; or Opioids and other non-opioid analgesics

**Abbreviations**: NSAIDs (nonsteroidal anti-inflammatory drugs), CGRP (Calcitonin gene-related peptide)

### Monotherapy drug persistence

Consistent with the above information, where patients do not return to outpatient consultation for migraine and do not return to health services to claim medication, the duration of first-line^1^ treatment was studied for both preventive and acute therapy.

A total of 71.8% of the patients received any first-line preventive treatment, most of whom started treatment with propranolol (*n* = 32,721), valproic acid (*n* = 6,828) or amitriptyline (*n* = 5,963). However, the drugs with the greatest mean duration in days were divalproex sodium (*n* = 782), followed by onabotulinum toxin A (*n* = 2,078) and topiramate (*n* = 5,843), with 247.1 (SD: 308.5), 221.4 (SD: 291.1) and 183.2 (SD: 231.7) days, respectively. Overall, the survival analysis of patients who discontinued treatment until they switched medication tended to last 273 days (95% CI: 266–280), and when examined via subgroup analysis, it can be seen that patients who discontinued treatment had the longest median duration of 311 days (95% CI: 304–326), and those who started treatment with antipsychotics had the shortest median duration of 85 days (95% CI: 16-NA) (Fig. 2S).

On the other hand, 57.9% of the patients were observed to have first-line acute therapy^2^, 43.4% of whom were treated with NSAIDs (*n* = 32,257), and 0.3% of whom were treated with corticosteroids (*n* = 190). It is also worth noting that 1.4% (*n* = 1.053) of those patients who started with opioids were affected. In addition, zolmitriptan (*n* = 639) had the longest duration, with a mean of 309.7 days (SD: 339.2), and the shortest mean durations were for dehidrocodeine (*n* = 48) and prednisolone (*n* = 190), with 113.8 and 125.4 days, respectively. However, the survival analysis until treatment switch indicated a fixed overall median duration of approximately 90 days (95% CI 90–90), whereas the subgroup analysis indicated that triptans had the longest median duration of 267 days (95% CI 252–283), and the other treatments had a median duration of 90 days (95% CI 90–90) (Fig. 3S).

### Use of health resources

**Fig. 4:**
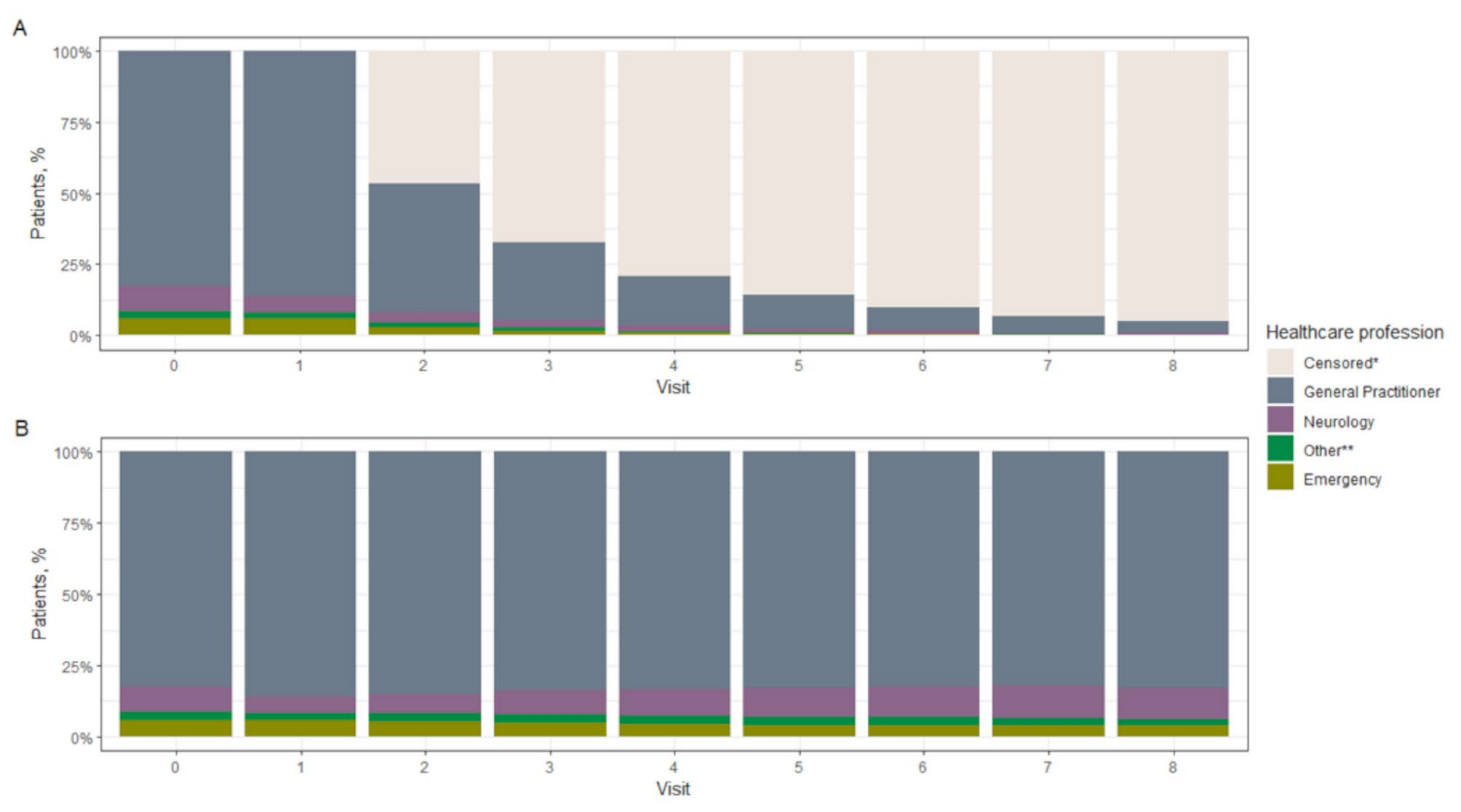
Utilization of the health professional group in the initial diagnosis and follow-up. **A)** all migraine population; **B)** percentage of specialists according to health care. * Censored (patients with no new health care during subsequent follow-up). ** Other: Internists, family physician, occupational physician, anesthetists, Gynecologists, Nephrologists, Ophthalmologists, Otorhinolaryngologists, Psychiatrist/psychotherapist, General surgery, Endocrinologists, Neurosurgery, Bioenergetics, dramatology, physiatry, gastroenterology, and allergology

Across the 5-year study period, the mean follow-up period was 3.7 years (SD: 1.2), and within this timeframe, the vast majority of patients were first seen by a general practitioner (82.6%, 73,657/89,227), and only 8.9% of patients (7,901/89,227) were first seen by a neurologist (first visit). Approximately 5.7% (5,053/89,227) of patients visited an emergency specialist. During the follow-up visits, for patients who continued to go to visits until the last observed visit, patients seen by a neurologist represented a greater share (11%, 458/4,274), and visits to emergency specialists decreased to 3.6% (155/4,274) (Fig. 4, Part b).

For each year of follow-up, the mean numbers of outpatient visits and hospitalizations were 0.68 (SD 1.0) and 0.001 (SD 0.025), respectively. Regarding the use of health care resources per patient during the follow-up period, a mean of 2.9 (SD 1.82) outpatient visits with a general practitioner and 0.54 (SD 0.94) with a specialist were observed. Overall, a mean of 0.19 (SD 0.64) of patients received emergency health care, and a mean of 0.11 (SD 0.33), 0.07 (SD 0.29) and 0.008 (SD 0.09) received computed tomography, magnetic resonance imaging and electroencephalography, respectively.

## Discussion

The results of this study, based on health electronic databases, show that the prevalence of migraine in Colombia from 2018 to 2022 varies between 1.69 and 2.42 patients in 100 affiliates, which is lower than that reported in other studies conducted in Latin America or Colombia. A wide variability in prevalence was found in previous studies conducted in Colombia [15–17]. Pradilla was found to have a prevalence of 7.1% (95% CI 6.5–7.7) in 1995–1996 using clinical examination based on World Health Organization protocols [15]. Morillo conducted a survey using the criteria of 1988 IHS in different countries, such as Colombia, in 1999, where the prevalence in females was 14.2% (95% CI 11.9–16.5) and that in males was 5.0% (95% CI 3.4–6.6) [16]. The most recent study was by Rueda [17], who also conducted another survey in a Colombian city using the Diagnostic Questionnaire for Migraine scale and reported a prevalence of 13.7% (95% CI 11.8–15.6). This heterogeneity is possibly related to the form of the prevalence estimation that depends on the study groups (such as hospital population and young people) or epidemiologists with interest in this disease, method used (e.g., nature of the screening question, number of conditions investigated, sampling method, number of participants), and case definition [18, 19]. This study provides real-world evidence using the electronic health databases of one insurance company with approximately 5 million affiliates. Russell et al. defined it as administrative prevalence, which suggests that its variability, compared to that of populational studies, is caused by self-medication of patients who did not see a health care professional after initial diagnosis or who were able to treat their episodes due to the low frequency of attacks [13]. Additionally, according to a study by Eurolight in Europe, 15.8–33% of the population with migraine consulted a health care professional [20], and Morillo reported that 50.9% of patients did not consult a health care professional [16].

The incidence of migraine in Colombia was similar to that estimated by the Global Burden of Disease Study in 2019, in which for Andean Latin America, the age-standardized rate was 0.9 (95% CI 0.7–1.2) per 100,000 individuals aged 15–39 years, and that for Central Latin America was 1.3 (95% CI 1.1–1.7) per 100,000 individuals [19].

Both the incidence and prevalence were higher overall in the female and young adult age groups (18–24 and 25–34 years). This finding is similar to that described by Pacheco et al. [4] in a systematic review, which suggested that females had a four-fold greater chance of having migraine than males did (12.78%, 95% CI: 9.19–16.85 vs. 3.50%, 95% CI: 2.47–4.69). However, Morillo reported that the prevalence was higher in individuals aged between 30 and 39 years. 40–49 and 50–59 [16]. Pradilla reported the highest frequency of migraine between the third and fourth decades of life [15]. Other studies suggest that the prevalence increases in the young adult population until approximately age 40, then the prevalence starts to decline, which is similar to the trend observed in this study [21].

In contrast with a previous review [19], we found a decrease in the incidence of migraine since 2020, which may be related to environmental factors, strict diagnostic criteria, or patient preference for not consulting health care professionals during the COVID-19 pandemic. It has been argued that governmental measures related to the COVID-19 pandemic, such as the impact of lockdown on patients with migraine, may be modified by lifestyle and behavioral factors, as well as transformed or limited by healthcare [22]. However, currently published studies on headache in the setting of COVID-19 have concluded that some outcomes may increase or decrease the burden of disease, considering that headache-attributed burden not only has multiple and diverse components but is also very unevenly distributed in populations with current migraine [18, 22].

The frequency of cardiovascular conditions in migraine patients observed in this study was higher than 20% of the population due to essential hypertension. Additionally, the prevalence of hypercholesterolaemia and diabetes mellitus is between 5% and 10% in this population. Some studies have suggested that hypertension, dyslipidemia, and diabetes mellitus are considered risk factors for migraine [23]; however, these studies have yielded conflicting results, and the associations have not been validated [21, 24, 25]. The frequency of cardiovascular disease was similar to that reported in other studies in the United States [7] and Israel [26]. According to the Dodick study, which has similar data sources to those used in this study, the main cardiovascular diseases were ischemic cerebrovascular disease, structural heart disease, arrhythmias, uncontrolled hypertension, and ischemic heart disease, which accounted for less than 6.8% of the population [7]. On the other hand, these medical conditions are considered warnings for migraine patients, especially those receiving triptans, where treatments are limited to NSAIDs, nonopioid analgesics, and caffeine analgesic combinations [7]. However, the long-term use of NSAIDs increases the risk of myocardial infarction and stroke, hypertension, peptic ulcer disease and bleeding, and nephrotoxicity, especially in older adults [27–29].

According to the study, NSAIDs, corticosteroids and combinations of other analgesics were the main medications used for acute treatment. Triptans were used in lower proportions, but their use increased over time. This behavior is analogous to that observed in the study by Roessel et al. that was conducted in Germany, where NSAIDs were also more frequent, but they were characterized by ibuprofen and metamizole. Additionally, in the same study, corticosteroids were used as emergency medications [13]. In studies conducted in the United States, triptans were used more than to manage episodic migraine in more than 50% of patients, and rizatriptan and sumatriptan were the most commonly used triptans [30, 31].

Opioids were used in lower proportions in this study than in previous studies in Germany (19.1%) and the United States (8.3 − 53%); however, opioid use for more than 90 days was observed in more than 40% of patients. Another study in Colombia reported that almost 7% of patients were managed with tramadol [32], which is very similar to what was observed in this study. These findings are relevant given that the evidence of the efficacy of opioids is low or insufficient and that opioids are associated with increased rates of gastrointestinal-related adverse events, addiction, and drug abuse secondary to the treatment of migraine [33, 34]. For that reason, guidelines recommend against the use of opioids in the management of acute migraine [35, 36]; nevertheless, the use of opioids was observed in a high number of patients. Moreover, the use of opioids for acute migraine therapy after other analgesics or chronic use can indicate that patients need more efficacious treatment for migraine. Additionally, the persistence of episodic migraine was low during the follow-up period. NSAIDs were switched with another treatment in more than 50% of patients at 90 days of treatment and in more than 80% of patients during the year of follow-up. There was a lower proportion of patients who switched from treatments with triptans than other analgesics, but 50% of patients changed at 267 days, and almost 60% changed at the year of follow-up. This low persistence is another index to indicate the unmet needs of patients for more efficacious treatments, as mentioned before.

For preventive medications, the findings of the German population study by Roessler et al. were similar to those for beta-blockers, but metoprolol was the most frequent. Additionally, the use of anticonvulsants was also lower than that in the Colombian cohort, where antidepressants stand out. The use of onabotulinum toxin A was reported in only 0.8% of patients [13]. In the Ford et al. study, preventive treatment was used for 52% of episodic migraine patients and 95.6% of chronic migraine patients, where topiramate was the most frequent. Beta-blockers were reportedly used in only 8.0% of the population [31]. Woolley et al. reported that 65% of migraine patients did not receive preventive medications. Topiramate was the most frequent follow by beta-blocker and tricyclic antidepressants [30]. Moreover, the persistence of preventive medications was greater in the Colombian cohort, in which the median duration until patients required a switch was greater than 180 days, while in the Woolley cohort, it was 90 days [30].

Another common treatment has been ergotamine with its different combinations, mainly caffeine. According to this study, it was used in 20.5% in first visit with decreasing trend for subsequential visits. Based on a Colombian study that analyzed the appropriate use of ergotamine according to guidelines and medical interactions found that the 98.5% of prescription were unappropriated [37].

Another study conducted in Colombia in a cohort of 241 patients revealed that propranolol was the most commonly used treatment, followed by valproic acid and amitriptyline [38], which followed the same trend as the one in this study. Both Osorio et al. and this study observed treatments recommended by the American Headache Society (AHS) and the American Academy of Neurology (AAN), where topiramate, valproic acid, metoprolol, and propranolol are recommended for preventive treatment, and are supported by a high level of evidence [39]. CGRP monoclonal antibodies have been reported in very low proportions, possibly because of their recent availability on the Colombian market and their high cost compared to other treatments. These medications have been studied in Colombian patients under common practice assessing the clinical outcomes, however, the sample size was small [40]. Another important treatment is onabotulinum toxin A, which affects a wide group of patients every year and has adequate persistence.

This study intended to evaluate the persistence of acute and preventive treatment which vary between 85 and 311 days depending on treatment and other clinical patient characteristics. Persistence could consider a gross measure of effectiveness, however, it could be affected by other characteristics. An study conducted in Portugal evaluating the efficacy of preventive treatment found that it was 40% which was decreasing in successive prophylactic attempts. Additionally, it observed high percentage of dropout [41].

According to this study, the management of patients with migraine is mainly conducted by general practitioners, and a low proportion of patients with migraine are managed by neurologists or emergency specialists. This finding is similar to that of a previous study in Colombia by Morillo [16], which was conducted in 1999, implying that the management of migraines did not undergo major changes after 20 years. In Germany, the results of this study were very similar to those of other studies in which management was led by a general practitioner; however, neurologists participated more than in Colombia [13]. However, in a United States cohort, neurologists were more involved in the management of migraine than in the Colombian cohort, and managed 30% of patients with episodic migraine and 65% of patients with chronic migraine [31]. However, the number of consultations was very similar to that in Colombia, where there were 1.9 to 2.8 days of consultations per year. The frequency of hospitalization was 3.6% according to Ford et al. [31], but in this study, it was 0.1%.

This study is the first in Colombia to analyze electronic health databases, including medical records, claim databases and laboratories. One strength of this study is that it can analyze a large number of patients from almost 20% of the contributive regime in Colombia, which increases the representativity of the Colombian population. The information of the HMO allows us to register all care that affiliates receive, including the supply of treatment, hospitalization, or medical consultation. The patients who were selected for inclusion in the analysis were confirmed by physicians or clinical specialists, which decreased misclassification bias and allowed us to guarantee the accurate characterization of patients with migraine. Additionally, five years of analysis of the cohort was feasible for the organization and management of the data by the HMO; thus, any variation in the cohort during the follow-up made it possible to analyze and decrease any bias due to secular tendencies.

Our study is the most comprehensive to report on migraine epidemiology in Colombia. Although it does not reflect the true prevalence of the disease, this study provides valuable information for clinicians and national policy-makers, presenting the burden of migraine in the health care system and providing a basis for decisions on health resource allocation.

This study has some limitations due to the nature of the study and the availability of information. The first filter of the study was limited by the use of the ICD-10, in which patients for whom the physician was not adequately registered were excluded from the study. The sources of data used in this study are widely used and validated for reporting the outcomes described. However, databases have limited records since they can suffer from omissions, site-specific coding problems, coding bias, incorrect coding, and insufficient detail in the characterization of disease chronicity. This was mitigated with the parameterized collection of the variables established in the protocol. The options for the variables were predefined in accordance with the availability and structure of the site data. Another limitation is the risk of patients having a combination of episodic and chronic migraine, given that patients can be diagnosed by other insurance companies and that insurance companies can change during follow-up. There was an important group of patients who were lost to follow-up given that they claimed treatment and that there was no registration of other care for other episodes of migraine in the health care system. Finally, patients who were self-medicated or had migraines without a diagnosis were not included in this study, reducing the representativeness of the study.

## Conclusions

The prevalence of migraine in Colombia according to health electronic databases was lower than that reported in previous studies conducted in the country, suggesting that many migraine patients manage the disease outside of the health care system. Additionally, the treatment patterns for acute and preventive treatment of migraine follow the recommendations of different guidelines; however, the low persistence, high use of opioids, and comorbidities of patients, especially cardiovascular disease, are relevant aspects to evaluate in the management of migraine. Finally, the participation of neurologists in the management of migraine patients was very low, and increasing the involvement of neurologists in migraine management will improve the effectiveness of therapy, considering the conditions and characteristics of patients with migraine.

## Electronic supplementary material

Below is the link to the electronic supplementary material.

Supplementary Material 1

Supplementary Material 2

## Data Availability

The data that support the findings of this study are available from SURAMERICANA HMO but restrictions apply to the availability of these data, which were used under agreement for the current study with Pfizer SAS, and so are not publicly available.

## Footnotes

1 In this case, considering the limitations of administrative records, a line of treatment is understood here as consecutive deliveries of the same medicine, across the 15 observed claims records.

2 Excluding dexamethasone and combinated therapies.
