# Supplementary material for: Epidemiological, clinical characterization and treatment patterns of migraine patients in a Colombian cohort from 2018 to 2022
Source: J Headache Pain. 2024 Dec 24;25(1):226. doi: 10.1186/s10194-024-01918-9 (PMC11667797; doi:10.1186/s10194-024-01918-9)
Supplement: Supplementary file 1 — Supplementary Material 1 [file 10194_2024_1918_MOESM1_ESM.docx]

| **Supplementary Table 1. Special interest diagnosis: cardiovascular risk factor and ICD-10 code** |
| --- |
| **Ischemic cerebrovascular disease**  ≥1 inpatient, emergency department, or outpatient claim with diagnosis code in any position in 12-month pre-period*  G45 Transient cerebral ischemic attacks and related syndromes  G46 Vascular syndromes of brain in cerebrovascular diseases  I63 Cerebral infarction  I65 Occlusion and stenosis of precerebral arteries, not resulting in cerebral infarction  I66 Occlusion and stenosis of cerebral arteries, not resulting in cerebral infarction  I67.82 Cerebral ischemia  I69.3 Sequelae of cerebral infarction  Z86.73 Personal history of transient ischemic attack (TIA), and cerebral infarction w/o residual deficits  **Ischemic heart disease**  ≥1 inpatient, emergency department, or outpatient claim with diagnosis code in any position in 12-month pre-period*  I20 Angina pectoris  I21 ST elevation (STEMI) and non-ST elevation (NSTEMI) myocardial infarction  I22 Subsequent ST elevation (STEMI) and non-ST elevation (NSTEMI) myocardial infarction  I23 Certain current complications following ST elevation (STEMI) and non-ST elevation (NSTEMI) myocardial infarction (within the 28 day period)  I24 Other acute ischemic heart diseases  I25 Chronic ischemic heart disease  Z95.1 Presence of aortocoronary bypass graft  Z95.5 Presence of coronary angioplasty implant and graft  Z98.61 Coronary angioplasty status  **Other cerebrovascular and cardiovascular disease**  Criteria for claims identification:  ≥1 inpatient or emergency department claim with diagnosis code in any position in 6-month pre-period**  I60 Nontraumatic subarachnoid hemorrhage  I61 Nontraumatic intracerebral hemorrhage  I62 Other and unspecified nontraumatic intracranial hemorrhage  Criteria for claims identification:  ≥1 inpatient, emergency department, or outpatient claim with diagnosis code in any position in 12-month pre-period*  I67 Other cerebrovascular diseases  (Excludes: I67.4 Hypertensive encephalopathy, I67.82 Cerebral ischemia)  I68.2 Cerebral arteritis in other diseases classified elsewhere  I68.8 Other cerebrovascular disorders in diseases classified elsewhere  I69 Sequelae of cerebrovascular disease  I45.6 Pre-excitation syndrome (Wolff-Parkinson-White syndrome)  E78.01 Familial hypercholesterolemia  **Peripheral artery disease**  ≥1 inpatient, emergency department, or outpatient claim with diagnosis code in any position in 12-month pre-period*  I70 Atherosclerosis  I73 Other peripheral artery diseases  I79.1 Aortitis in diseases classified elsewhere  I79.8 Other disorders of arteries, arterioles and capillaries in diseases classified elsewhere  E08.51 Diabetes mellitus due to underlying condition w/ diabetic peripheral angiopathy w/o gangrene  E08.52 Diabetes mellitus due to underlying condition w/ diabetic peripheral angiopathy w/ gangrene  E09.51 Drug or chemical induced diabetes mellitus w/ diabetic peripheral angiopathy w/o gangrene  E09.52 Drug or chemical induced diabetes mellitus w/ diabetic peripheral angiopathy w/ gangrene  E10.51 Type 1 diabetes mellitus w/ diabetic peripheral angiopathy w/o gangrene  E10.52 Type 1 diabetes mellitus w/ diabetic peripheral angiopathy w/ gangrene  E11.51 Type 2 diabetes mellitus w/ diabetic peripheral angiopathy w/o gangrene  E11.52 Type 2 diabetes mellitus w/ diabetic peripheral angiopathy w/ gangrene  E13.51 Other specified diabetes mellitus w/ diabetic peripheral angiopathy w/o gangrene  E13.52 Other specified diabetes mellitus w/ diabetic peripheral angiopathy w/ gangrene  Z95.820 Peripheral vascular angioplasty status w/ implants and grafts  Z98.62 Peripheral vascular angioplasty status    **Uncontrolled hypertension**  ≥1 inpatient, emergency department, or outpatient claim with diagnosis code in any position in 12-month pre-period*  I10 Essential (primary) hypertension  I11 Hypertensive heart disease  I12 Hypertensive chronic kidney disease  I13 Hypertensive heart and chronic kidney disease  I15 Secondary hypertension  I16.0 Hypertensive urgency  I16.1 Hypertensive emergency  I16.9 Hypertensive crisis, unspecified  I67.4 Hypertensive encephalopathy  H35.03 Hypertensive retinopathy    **Gastrointestinal ischemia**  ≥1 inpatient, emergency department, or outpatient claim with diagnosis code in any position in 12-month pre-period*  D73.5 Infarction of spleen  K55.0 Acute vascular disorders of intestine    **Cardiac surgery and/or implants**  Z95.2 Presence of prosthetic heart valve  Z95.3 Presence of xenogenic heart valve  Z95.4 Presence of other heart-valve replacement  Z95.810 Presence of automatic (implantable) cardiac defibrillator  Z95.811 Presence of heart assist device  Z95.812 Presence of fully implantable artificial heart  Z95.818 Presence of other cardiac implants and grafts  Z95.828 Presence of other vascular implants and grafts  Z95.9 Presence of cardiac and vascular implant and graft, unspecified    **Structural heart disease**  I05 Rheumatic mitral valve diseases  I06 Rheumatic aortic valve diseases  I07 Rheumatic tricuspid valve diseases  I08 Multiple valve diseases  I09 Other rheumatic heart diseases  I34 Nonrheumatic mitral valve disorders  I35 Nonrheumatic aortic valve disorders  I36 Nonrheumatic tricuspid valve disorders  I37 Nonrheumatic pulmonary valve disorders  I38 Endocarditis, valve unspecified  I39 Endocarditis and heart valve disorders in diseases classified elsewhere  I42.1 Obstructive hypertrophic cardiomyopathy  I42.2 Other hypertrophic cardiomyopathy  I50 Heart failure  Q20 Congenital malformations of cardiac chambers and connections  Q21 Congenital malformations of cardiac septa  Q22 Congenital malformations of pulmonary and tricuspid valves  Q23 Congenital malformations of aortic and mitral valves  Q24 Other congenital malformations of heart  Q25 Congenital malformations of great arteries  Q26 Congenital malformations of great veins  **Arrhythmia**  I44.1 Atrioventricular block, second degree  I44.2 Atrioventricular block, complete  I44.7 Left bundle-branch block, unspecified  I45.3 Trifascicular block  I45.81 Long QT syndrome  I45.89 Other specified conduction disorders  I47.0 Re-entry ventricular arrhythmia  I47.1 Supraventricular tachycardia  I47.2 Ventricular tachycardia  I47.9 Paroxysmal tachycardia, unspecified  I48.0 Paroxysmal atrial fibrillation  I48.1 Persistent atrial fibrillation  I48.2 Chronic atrial fibrillation  I48.3 Typical atrial flutter  I48.4 Atypical atrial flutter  I48.91 Unspecified atrial fibrillation  I48.92 Unspecified atrial flutter  I49.01 Ventricular fibrillation  I49.02 Ventricular flutter  I49.5 Sick sinus syndrome  I49.8 Other specified cardiac arrhythmias  I49.9 Cardiac arrhythmia, unspecified  **Other cardiac conditions**  I46 Cardiac arrest  I71 Aortic aneurysm and dissection  I74 Arterial embolism and thrombosis  R55 Syncope and collapse  R94.30 Abnormal result of cardiovascular function study, unspecified  R94.39 Abnormal result of other cardiovascular function study  Z82.41 Family history of sudden cardiac death  Z86.711 Personal history of pulmonary embolism  Z86.718 Personal history of other venous thrombosis and embolism  Z86.74 Personal history of sudden cardiac arrest |

| **Supplementary Table 2. Characteristics of population with migraine** | | | | | | | | | | | | | | | |
| --- | --- | --- | --- | --- | --- | --- | --- | --- | --- | --- | --- | --- | --- | --- | --- |
|  | | **Type** | | | | | | | | | | | | **Total**  **n=(89,227)** | |
| **Diagnosis to date index** | | **G43.0 Migraine without aura (common migraine)**  **n=(** **10,166)** | | **G43.1 Migraine with aura (classical migraine)**  **n=** (**5,594)** | | **G43.2 Status migrainosus**  **n=(** **4,512)** | | **G43.3 Complicated migraine (including chronic migraine)**  **n=(10,666)** | | **G43.8 Other migraine**  **n=(14,542)** | | **G43.9 Migraine, unspecified**  **n=(43,747)** | |  |  |
|  |  | **n** | **%** | **n** | **%** | **n** | **%** | **n** | **%** | **n** | **%** | **n** | **%** | **n** | **%** |
| **Sex** | Female | 8,710 | 85.7 | 4,751 | 84.9 | 3,670 | 81.3 | 8,337 | 78.2 | 12,499 | 86.0 | 37,759 | 86.3 | 75,726 | 84.9 |
|  | Male | 1,456 | 14.3 | 843 | 15.1 | 842 | 18.7 | 2,329 | 21.8 | 2,043 | 14.0 | 5,988 | 13.7 | 13,501 | 15.1 |
| **Age, total** | 18-24 | 1,990 | 19.6 | 1,090 | 19.5 | 716 | 15.9 | 796 | 7.5 | 3,092 | 21.3 | 9,234 | 21.1 | 16,918 | 19.0 |
|  | 25-34 | 3,381 | 33.3 | 2,068 | 37.0 | 1,651 | 36.6 | 1,882 | 17.6 | 5,268 | 36.2 | 15,836 | 36.2 | 30,086 | 33.7 |
|  | 35-44 | 2,214 | 21.8 | 1,257 | 22.5 | 927 | 20.5 | 1,928 | 18.1 | 3,174 | 21.8 | 9,580 | 21.9 | 19,080 | 21.4 |
|  | 45-54 | 1,491 | 14.7 | 690 | 12.3 | 498 | 11.0 | 2,001 | 18.8 | 1,866 | 12.8 | 5,627 | 12.9 | 12,173 | 13.6 |
|  | 55-64 | 728 | 7.2 | 297 | 5.3 | 334 | 7.4 | 1,811 | 17.0 | 852 | 5.9 | 2,475 | 5.7 | 6,497 | 7.3 |
|  | 65-74 | 280 | 2.8 | 119 | 2.1 | 228 | 5.1 | 1,181 | 11.1 | 228 | 1.6 | 750 | 1.7 | 2,786 | 3.1 |
|  | ≥ 75 Years | 82 | 0.8 | 73 | 1.3 | 158 | 3.5 | 1,067 | 10.0 | 62 | 0.4 | 245 | 0.6 | 1,687 | 1.9 |
| **Type of health insurance regime ^a^** | Contributive | 9,318 | 91.7 | 5,117 | 91.5 | 4,177 | 92.6 | 10,035 | 94.1 | 13,308 | 91.5 | 39,904 | 91.2 | 81,859 | 91.7 |
|  | Subsidized | 848 | 8.3 | 477 | 8.5 | 335 | 7.4 | 631 | 5.9 | 1,234 | 8.5 | 3,843 | 8.8 | 7,368 | 8.3 |
| ^a^ Type of affiliation to the General System of Social Security in Health (SGSSS in Spanish abbreviation) in Colombia: Contributive regime (it provides mandatory coverage to workers in the formal sector), and Subsidized regime (it covers the low-income population that does not have the capacity to contribute to health system).  **Abbreviations:** n, number of patients | | | | | | | | | | | | | | | |

| **Supplementary Table 3. Prevalence (%*) of migraine population by age** | | | | | | | | | | | | | | | |
| --- | --- | --- | --- | --- | --- | --- | --- | --- | --- | --- | --- | --- | --- | --- | --- |
|  | **2018** | | | **2019** | | | **2020** | | | **2021** | | | **2022** | | |
|  | **N** | **Product** | **95%CI** | **N** | **Product** | **95%CI** | **N** | **Product** | **95%CI** | **N** | **Product** | **95%CI** | **N** | **Product** | **95%CI** |
| 18-24 | 8,145 | 2.96 | 2.9 - 3.03 | 12,987 | 3.93 | 3.86 - 3.99 | 15,717 | 4.03 | 3.97 - 4.1 | 15,965 | 3.58 | 3.52 - 3.63 | 16,907 | 3.31 | 3.26 - 3.36 |
| 25-34 | 14,518 | 2.46 | 2.43 - 2.51 | 23,221 | 3.40 | 3.36 - 3.45 | 28,232 | 3.42 | 3.38 - 3.46 | 28,589 | 3.03 | 3 - 3.07 | 30,045 | 3.01 | 2.97 - 3.04 |
| 35-44 | 9,121 | 1.57 | 1.53 - 1.6 | 14,676 | 2.23 | 2.2 - 2.27 | 17,788 | 2.32 | 2.29 - 2.36 | 18,042 | 2.14 | 2.11 - 2.17 | 19,049 | 2.16 | 2.13 - 2.19 |
| 45-54 | 6,087 | 1.48 | 1.45 - 1.52 | 9,442 | 2.01 | 1.97 - 2.05 | 11,292 | 2.07 | 2.03 - 2.11 | 11,414 | 1.91 | 1.88 - 1.94 | 12,117 | 1.90 | 1.87 - 1.94 |
| 55-64 | 2,999 | 0.84 | 0.81 - 0.87 | 4,729 | 1.13 | 1.10 - 1.17 | 5,833 | 1.22 | 1.19 - 1.25 | 5,850 | 1.12 | 1.09 - 1.15 | 6,395 | 1.13 | 1.1 - 1.15 |
| 65-74 | 1,179 | 0.59 | 0.56 - 0.63 | 1,940 | 0.82 | 0.78 - 0.86 | 2,475 | 0.93 | 0.89 - 0.97 | 24,21 | 0.84 | 0.81 - 0.88 | 2,664 | 0.84 | 0.81 - 0.87 |
| ≥ 75 years | 743 | 0.66 | 0.61 - 0.7 | 1,201 | 0.89 | 0.84 - 0.94 | 1,590 | 1.08 | 1.03 - 1.13 | 1,337 | 0.88 | 0.84 - 0.93 | 1,349 | 0.80 | 0.76 - 0.85 |
| *Period prevalence (Factor x 100) N = Observed cases of migraine CI =Confidence interval | | | | | | | | | | | | | | | |

| **Supplementary Table 4. Incidence* of migraine population by age** | | | | | | | | | | | | |
| --- | --- | --- | --- | --- | --- | --- | --- | --- | --- | --- | --- | --- |
|  | **2019** | | | **2020** | | | **2021** | | | **2022** | | |
|  | **N** | **Product** | **95%CI** | **N** | **Product** | **95%CI** | **N** | **Product** | **95%CI** | **N** | **Product** | **95%CI** |
| 18-24 | 4843 | 1.51 | 1.47 – 1.56 | 2728 | 0.73 | 0.70 – 0.76 | 257 | 0.06 | 0.053 - 0.07 | 945 | 0.19 | 0.18 – 0.20 |
| 25-34 | 8703 | 1.31 | 1.29 – 1.34 | 5004 | 0.63 | 0.61 – 0.64 | 395 | 0.043 | 0.039 - 0.05 | 1466 | 0.15 | 0.14 – 0.16 |
| 35-44 | 5557 | 0.86 | 0.84 – 0.89 | 3105 | 0.41 | 0.40 – 0.43 | 279 | 0.034 | 0.03 - 0.038 | 1018 | 0.12 | 0.11 – 0.13 |
| 45-54 | 3355 | 0.73 | 0.70 – 0.75 | 1841 | 0.34 | 0.33 – 0.36 | 171 | 0.029 | 0.025 - 0.03 | 719 | 0.12 | 0.11 – 0.12 |
| 55-64 | 1735 | 0.42 | 0.40 – 0.44 | 1090 | 0.23 | 0.22 – 0.24 | 88 | 0.017 | 0.014 - 0.021 | 585 | 0.10 | 0.10 – 0.11 |
| 65-74 | 767 | 0.33 | 0.30 – 0.35 | 518 | 0.20 | 0.18 – 0.21 | 26 | 0.009 | 0.006 - 0.013 | 296 | 0.09 | 0.08 – 0.11 |
| ≥ 75 years | 476 | 0.36 | 0.33 – 0.39 | 321 | 0.22 | 0.20 – 0.25 | 2 | 0.001 | 0.00 - 0.005 | 145 | 0.09 | 0.07 – 0.10 |
| *Migraine incidence rate (Factor x 100) N = Incident case CI =Confidence interval | | | | | | | | | | | | |

| **Supplementary Table 5. Concurrent diagnoses and cardiovascular risk factors in study population with migraine from database 2018 to 2022** | | | | | | |
| --- | --- | --- | --- | --- | --- | --- |
| **ICD-10 code** | **Diagnosis ^a^** | **Age categories (years)** | | | | **Overall**  **(n=** **89,227)**  **%** |
|  |  | **18 – 34**  **(n=** **47,004)**  **%** | **35 – 54**  **(n=** **31,253)**  **%** | **55 – 74**  **(n=** **9,283)**  **%** | **≥ 75 Years**  **(n=** **1,687)**  % |  |
| I10X | I10 Essential (primary) hypertension/Uncontrolled hypertension ^b, c^ | 7.3 | 26.6 | 61.8 | 88.3 | 21.3 |
| E78.0 | Pure hypercholesterolaemia | 2.3 | 9.7 | 20.8 | 11.1 | 7.0 |
| E11.9 | Type 2 diabetes mellitus (Without complications) ^d^ | 1.7 | 6.9 | 21.8 | 39.7 | 6.3 |
| R55 | Syncope and collapse ^b^ | 3.8 | 3.7 | 5.4 | 10.3 | 4.1 |
| E10.9 | Type 1 diabetes mellitus (Without complications) ^d^ | 0.7 | 2.7 | 9.5 | 20.6 | 2.7 |
| I67 | Other cerebrovascular diseases ^b^ | 0.7 | 2.5 | 8.1 | 12.6 | 2.3 |
| I50 | Heart failure ^b, f^ | 0.5 | 1.7 | 7.7 | 23.1 | 2.1 |
| I49.9 | Cardiac arrhythmia, unspecified ^e^ | 1.3 | 2.0 | 4.4 | 8.7 | 2.0 |
| I25 | Chronic ischemic heart disease ^b, c^ | 0.3 | 1.4 | 7.2 | 18.9 | 1.7 |
| I20 | Angina pectoris ^b, c^ | 0.5 | 1.9 | 5.5 | 6.6 | 1.6 |
| E14.9 | Unspecified diabetes mellitus (Without complications) | 0.3 | 1.3 | 5.5 | 12.5 | 1.4 |
| I63 | Cerebral infarction ^b^ | 0.2 | 0.6 | 2.4 | 5.5 | 0.6 |
| G45 | Transient cerebral ischemic attacks and related syndromes ^b^ | 0.2 | 1.4 | 4.0 | 7.1 | 1.1 |
| E11.8 | Type 2 diabetes mellitus (With unspecified complications) ^d^ | 0.1 | 0.8 | 4.1 | 9.9 | 1.0 |
| E10.8 | Type 1 diabetes mellitus (With unspecified complications) ^d^ | 0.2 | 0.8 | 3.9 | 9.2 | 1.0 |
| I69 | Sequelae of cerebrovascular disease ^b^ | 0.2 | 0.7 | 3.0 | 7.0 | 0.8 |
| I21 | Acute myocardial infarction ^b, f^ | 0.1 | 0.6 | 2.8 | 7.5 | 0.7 |
| I48X | Atrial fibrillation and flutter | 0.2 | 0.5 | 2.2 | 8.9 | 0.7 |
| I74 | Arterial embolism and thrombosis ^b, c^ | 0.3 | 0.7 | 1.9 | 3.0 | 0.6 |
| I73 | Other peripheral artery diseases ^b^ | 0.3 | 0.8 | 1.5 | 1.4 | 0.6 |
| H35.0 | Background retinopathy and retinal vascular changes | 0.2 | 0.5 | 2.0 | 5.2 | 0.6 |
| I15 | Secondary hypertension | 0.5 | 0.4 | 0.6 | 0.8 | 0.5 |
| E11.6 | Type 2 diabetes mellitus (With other specified complications [Diabetic arthropathy, Neuropathic diabetic arthropathy]) | 0.1 | 0.5 | 2.2 | 5.0 | 0.5 |
| E13.9 | Other specified diabetes mellitus (Without complications) | 0.1 | 0.5 | 1.9 | 4.6 | 0.5 |
| I11 | Hypertensive heart disease | 0.1 | 0.4 | 1.3 | 4.1 | 0.4 |
| I11.0 | Hypertensive heart disease with heart failure | 0.1 | 0.4 | 1.7 | 6.2 | 0.5 |
| I11.9 | Hypertensive heart disease without heart failure | 0.1 | 0.7 | 4.9 | 15.5 | 1.0 |
| I12 | Hypertensive renal disease | 0.1 | 0.2 | 1.8 | 7.6 | 0.4 |
| E10.6 | Type 1 diabetes mellitus (With other specified complications [Diabetic arthropathy, Neuropathic diabetic arthropathy]) | 0.1 | 0.3 | 1.8 | 4.5 | 0.4 |
| I47.1 | Supraventricular tachycardia | 0.3 | 0.4 | 0.9 | 1.5 | 0.4 |
| I47.9 | Paroxysmal tachycardia, unspecified | 0.3 | 0.4 | 0.5 | 0.4 | 0.4 |
| E10.7 | Type 1 diabetes mellitus (With multiple complications) | 0.1 | 0.2 | 1.5 | 4.6 | 0.4 |
| E11.7 | Type 2 diabetes mellitus (With multiple complications) | 0.0 | 0.3 | 1.4 | 3.8 | 0.3 |
| E11.2 | Type 2 diabetes mellitus (With renal complications) | 0.0 | 0.2 | 1.2 | 5.2 | 0.3 |
| I49.8 | Other specified cardiac arrhythmias (Brugada syndrome, Long QT syndrome, Rhythm disorder) | 0.2 | 0.3 | 0.7 | 2.0 | 0.3 |
| E10.2 | Type 1 diabetes mellitus (With renal complications) | 0.1 | 0.2 | 1.0 | 4.3 | 0.3 |
| I34 | Nonrheumatic mitral valve disorders | 0.1 | 0.3 | 0.6 | 1.3 | 0.3 |
| I70 | Atherosclerosis | 0.0 | 0.2 | 1.5 | 4.1 | 0.3 |
| I71 | Aortic aneurysm and dissection | 0.1 | 0.3 | 1.3 | 1.8 | 0.3 |
| E10.4 | Type 1 diabetes mellitus (With neurological complications) | 0.0 | 0.2 | 0.6 | 1.4 | 0.2 |
| G46 | Vascular syndromes of brain in cerebrovascular diseases | 0.0 | 0.2 | 0.5 | 0.8 | 0.2 |
| Q21 | Congenital malformations of cardiac septa | 0.1 | 0.2 | 0.5 | 0.0 | 0.2 |
| I35 | Nonrheumatic aortic valve disorders | 0.1 | 0.2 | 0.8 | 2.31 | 0.2 |
| I48.0 | Paroxysmal atrial fibrillation | 0.0 | 0.1 | 0.9 | 4.4 | 0.2 |
| I48.2 | Chronic atrial fibrillation | 0.0 | 0.1 | 0.8 | 5.4 | 0.2 |
| E11.0 | Type 2 diabetes mellitus with hyperosmolarity | 0.0 | 0.2 | 0.8 | 1.8 | 0.2 |
| E10.5 | Type 1 diabetes mellitus (With peripheral circulatory complications) | 0.0 | 0.1 | 0.4 | 0.8 | 0.1 |
| E11.4 | Type 2 diabetes mellitus (With neurological complications) | 0.0 | 0.1 | 0.7 | 1.1 | 0.1 |
| E13.6 | Other specified diabetes mellitus (With other specified complications) | 0.1 | 0.2 | 0.3 | 0.4 | 0.1 |
| E14.8 | Unspecified diabetes mellitus (With unspecified complications) | 0.0 | 0.1 | 0.4 | 0.8 | 0.1 |
| I65 | Occlusion and stenosis of precerebral arteries, not resulting in cerebral infarction | 0.0 | 0.1 | 0.6 | 1.5 | 0.1 |
| I22 | Subsequent myocardial infarction | 0.0 | 0.0 | 0.3 | 0.5 | 0.1 |
| I48.1 | Persistent atrial fibrillation | 0.0 | 0.0 | 0.3 | 1.8 | 0.1 |
| Z95.5 | Presence of coronary angioplasty implant and graft | 0.0 | 0.1 | 0.9 | 3.1 | 0.2 |
| I05 | Rheumatic mitral valve diseases | 0.1 | 0.1 | 0.5 | 0.5 | 0.1 |
| I06 | Rheumatic aortic valve diseases | 0.0 | 0.1 | 0.2 | 0.4 | 0.1 |
| I60 | Nontraumatic subarachnoid hemorrhage | 0.1 | 0.2 | 0.3 | 0.4 | 0.1 |
| I61 | Nontraumatic intracerebral hemorrhage | 0.0 | 0.1 | 0.4 | 0.9 | 0.1 |
| I07 | Rheumatic tricuspid valve diseases | 0.0 | 0.1 | 0.1 | 0.4 | 0.1 |
| I44.1 | Atrioventricular block, second degree | 0.0 | 0.1 | 0.2 | 0.8 | 0.1 |
| I44.2 | Atrioventricular block, complete | 0.0 | 0.0 | 0.2 | 0.8 | 0.1 |
| I44.7 | Left bundle-branch block, unspecified | 0.0 | 0.1 | 0.4 | 0.5 | 0.1 |
| I49.0 | Ventricular fibrillation and flutter | 0.0 | 0.1 | 0.5 | 2.6 | 0.1 |
| I49.5 | Sick sinus syndrome | 0.0 | 0.1 | 0.3 | 0.9 | 0.1 |
| I47.2 | Ventricular tachycardia | 0.0 | 0.1 | 0.3 | 0.8 | 0.1 |
| I68.8 | Other cerebrovascular disorders in diseases classified elsewhere | 0.0 | 0.1 | 0.4 | 0.5 | 0.1 |
| Z95.2 | Presence of prosthetic heart valve | 0.0 | 0.1 | 0.5 | 0.4 | 0.1 |
| Z95.8 | Presence of other cardiac and vascular implants and grafts | 0.0 | 0.1 | 0.5 | 1.1 | 0.1 |
| Z95.9 | Presence of cardiac and vascular implant and graft, unspecified | 0.0 | 0.1 | 0.2 | 0.5 | 0.1 |
| I39 | Endocarditis and heart valve disorders in diseases classified elsewhere | 0.0 | 0.1 | 0.1 | 0.4 | 0.1 |
| Q25 | Congenital malformations of great arteries | 0.1 | 0.0 | 0.2 | 0.3 | 0.1 |
| I24 | Other acute ischemic heart diseases | 0.0 | 0.1 | 0.2 | 0.3 | 0.1 |
| I62 | Other and unspecified nontraumatic intracranial hemorrhage | 0.0 | 0.0 | 0.3 | 0.3 | 0.1 |
| I13 | Hypertensive heart and renal disease | 0.0 | 0.0 | 0.1 | 0.8 | 0.1 |
| I23 | Certain current complications following acute myocardial infarction | 0.0 | 0.0 | 0.2 | 0.6 | 0.0 |
| R94.3 | Abnormal results of cardiovascular function studies | 0.0 | 0.1 | 0.1 | 0.4 | 0.0 |
| I08 | Multiple valve diseases | 0.0 | 0.0 | 0.2 | 0.5 | 0.0 |
| I47.0 | Re-entry ventricular arrhythmia | 0.0 | 0.1 | 0.0 | 0.2 | 0.0 |
| E11.5 | Type 2 diabetes mellitus (With peripheral circulatory complications) | 0.0 | 0.0 | 0.1 | 0.7 | 0.0 |
| I66 | Occlusion and stenosis of cerebral arteries, not resulting in cerebral infarction | 0.0 | 0.0 | 0.1 | 0.0 | 0.0 |
| I37 | Nonrheumatic pulmonary valve disorders | 0.0 | 0.0 | 0.1 | 0.1 | 0.0 |
| Q22 | Congenital malformations of pulmonary and tricuspid valves | 0.0 | 0.0 | 0.1 | 0.0 | 0.0 |
| Q24 | Other congenital malformations of heart | 0.0 | 0.0 | 0.1 | 0.0 | 0.0 |
| I45.8 | Other specified conduction disorders | 0.0 | 0.0 | 0.1 | 0.3 | 0.0 |
| I46 | Cardiac arrest | 0.0 | 0.0 | 0.1 | 0.4 | 0.0 |
| I79.8 | Other disorders of arteries, arterioles and capillaries in diseases classified elsewhere | 0.0 | 0.0 | 0.1 | 0.1 | 0.0 |
| Z95.3 | Presence of xenogenic heart valve | 0.0 | 0.0 | 0.1 | 0.2 | 0.0 |
| Z95.4 | Presence of other heart-valve replacement | 0.0 | 0.0 | 0.1 | 0.0 | 0.0 |
| I42.2 | Other hypertrophic cardiomyopathy | 0.0 | 0.0 | 0.1 | 0.2 | 0.0 |
| Z86.6 | Personal history of diseases of the nervous system and sense organs | 0.0 | 0.0 | 0.0 | 0.0 | 0.0 |
| **^a^** Classification according to the diagnoses evidenced after the index date.  ^b^ Contraindication or special warning and precaution for use for Triptans  ^c^ Contraindication or special warning and precaution for use for Ergotamine derivatives  ^d^ Contraindication or special warning and precaution for use for Pregabalin  ^e^ Contraindication or special warning and precaution for use for Beta-blockers  ^f^ Contraindication or special warning and precaution for use for amitriptyline  **Abbreviations:** n, number of patients; n, number of patients; ICD-10, International Classification of Diseases, tenth revision | | | | | | |
