# Supplementary figures and images for: Epidemiological, clinical characterization and treatment patterns of migraine patients in a Colombian cohort from 2018 to 2022
Source: J Headache Pain. 2024 Dec 24;25(1):226. doi: 10.1186/s10194-024-01918-9 (PMC11667797; doi:10.1186/s10194-024-01918-9)

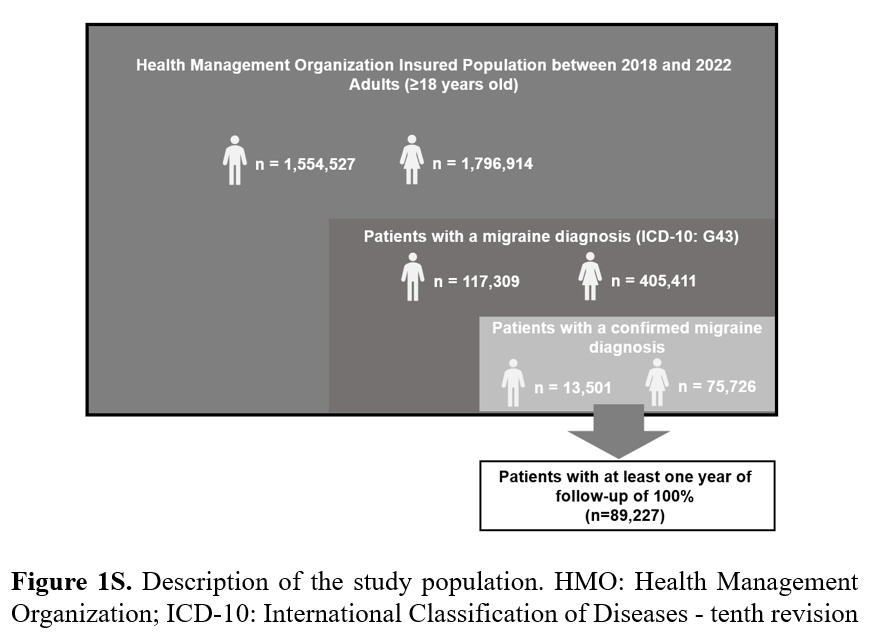

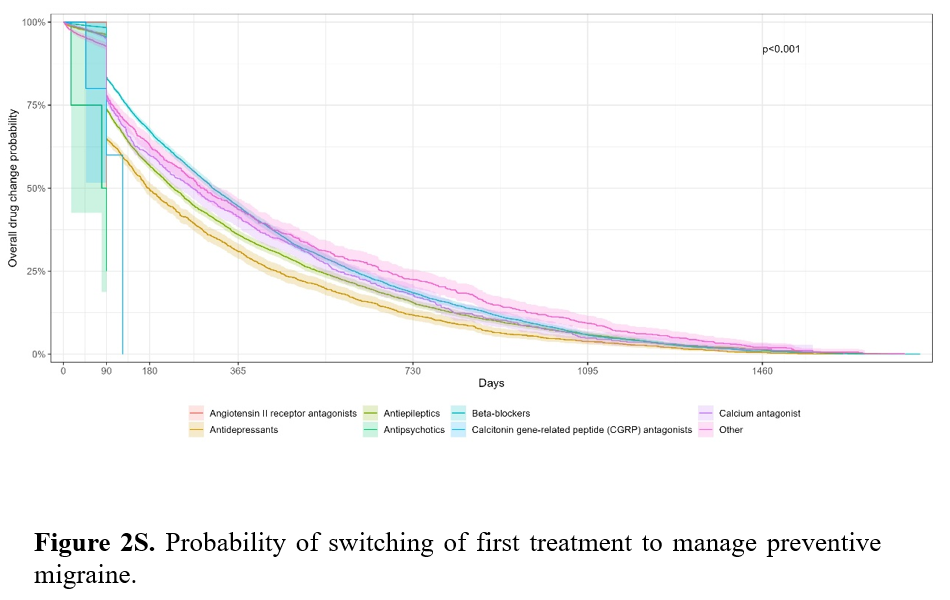

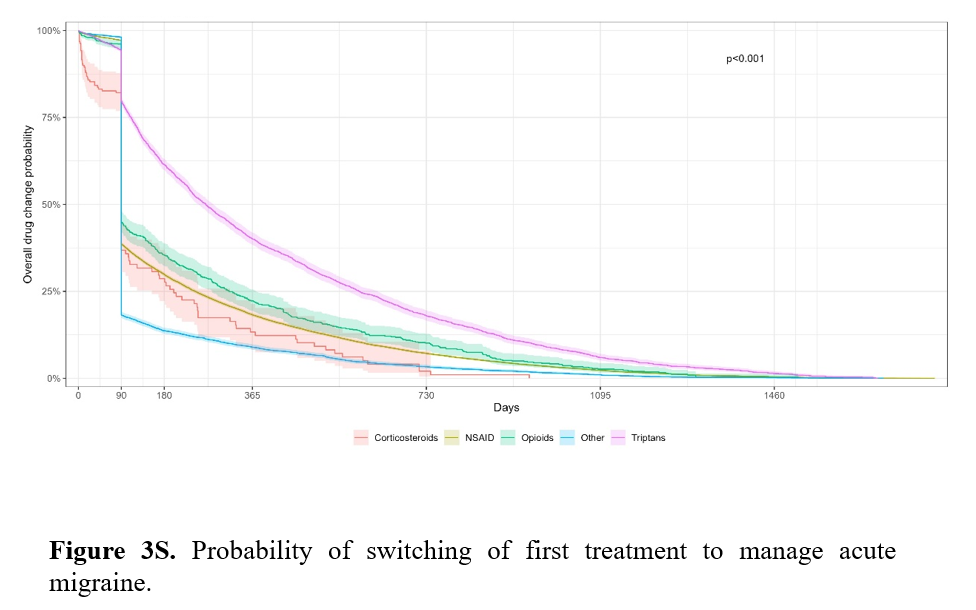

Supplement: Supplementary file 2 — Supplementary Material 2 [file 10194_2024_1918_MOESM2_ESM.docx]
